# Hydroxychloroquine-induced cardiomyopathy: role of cardiac magnetic resonance for the diagnosis and follow-up of a very rare entity—a case report

**DOI:** 10.1093/ehjcr/ytae404

**Published:** 2024-08-30

**Authors:** Lucía Cobarro Gálvez, Silvia Valbuena-López, Elsa Prieto Moriche, Elena Ruiz Bravo-Burguillos, Esther Pérez David

**Affiliations:** Cardiology Department, La Paz University Hospital, Paseo de la Castellana, 261, 28046 Madrid, Spain; Cardiology Department, La Paz University Hospital, Paseo de la Castellana, 261, 28046 Madrid, Spain; Cardiology Department, La Paz University Hospital, Paseo de la Castellana, 261, 28046 Madrid, Spain; Anatomical Pathology Department, La Paz University Hospital, Paseo de la Castellana, 261, 28046 Madrid, Spain; Cardiology Department, La Paz University Hospital, Paseo de la Castellana, 261, 28046 Madrid, Spain

**Keywords:** Cardiac MRI, Endomyocardial biopsy, Hydroxychloroquine, Hydroxychloroquine-induced cardiomyopathy, Infiltrative cardiomyopathy, Myocardial tissue characterization, Case report

## Abstract

**Background:**

Hydroxychloroquine (HCQ) is a disease-modifying antirheumatic used in rheumatological diseases such as systemic lupus erythematosus. Long-term exposure to HCQ results in drug accumulation and predisposes to adverse effects.

**Case summary:**

We present the case of a 45-year-old woman with long-term treatment with HCQ who presented to the Emergency Department with acute heart failure. Transthoracic echocardiogram, previously normal, showed severe biventricular hypertrophy and biventricular systolic dysfunction. Cardiac magnetic resonance (CMR) confirmed the previous findings and showed elevated native T1 and T2 values, elevated extracellular volume, and extensive mid-wall late gadolinium enhancement (LGE). Infiltrative cardiomyopathy was suspected, and endomyocardial biopsy performed. Light microscopy showed myocyte hypertrophy and vacuolar change and absence of lymphocytic inflammatory infiltrates. The diagnosis of HCQ-induced cardiomyopathy was established, and the drug was withdrawn. A CMR performed 1 year later showed normal systolic function of both ventricles and normalization of T2 values, reflecting resolution of myocardial oedema. However, severe hypertrophy, elevated native T1 values, and LGE persisted.

**Discussion:**

Our case shows that although discontinuation of the drug stops the progression of the disease, established myocardial structural damage persists. Early diagnosis of this entity is therefore essential to improve prognosis.

**Learning points**

- The diagnosis of antimalarial-induced cardiomyopathy is challenging: no specific sign of hydroxychloroquine cardiotoxicity is available, but cardiac magnetic resonance imaging can rule out other conditions such as Fabry disease or ischaemic heart disease.
- Endomyocardial biopsy remains necessary in most cases to establish the definite diagnosis.
- Early diagnosis is crucial to discontinue treatment with hydroxychloroquine and prevent disease progression.

## Introduction

Hydroxychloroquine (HCQ) has been a cornerstone in treating systemic lupus erythematosus (SLE) and other rheumatic diseases. Administered orally, this drug has high bioavailability, a large volume of distribution, and a long half-life (30–60 days).^1^ While retinal toxicity is one of the most feared adverse effects, HCQ can also cause cardiomyopathy. Early diagnosis of HCQ-induced cardiomyopathy, also known as antimalarial-induced cardiomyopathy (AMIC), is crucial to prevent fatal outcomes. Hydroxychloroquine-induced cardiac toxicity should be suspected if a patient on this drug develops ventricular dysfunction, prompting immediate discontinuation. Most cases reported in the literature present with a restrictive cardiomyopathy phenotype characterized by increased wall thickness, but cases of dilated cardiomyopathy have been reported.^2^ However, diagnosing AMIC remains challenging.

## Summary figure

**Figure:**
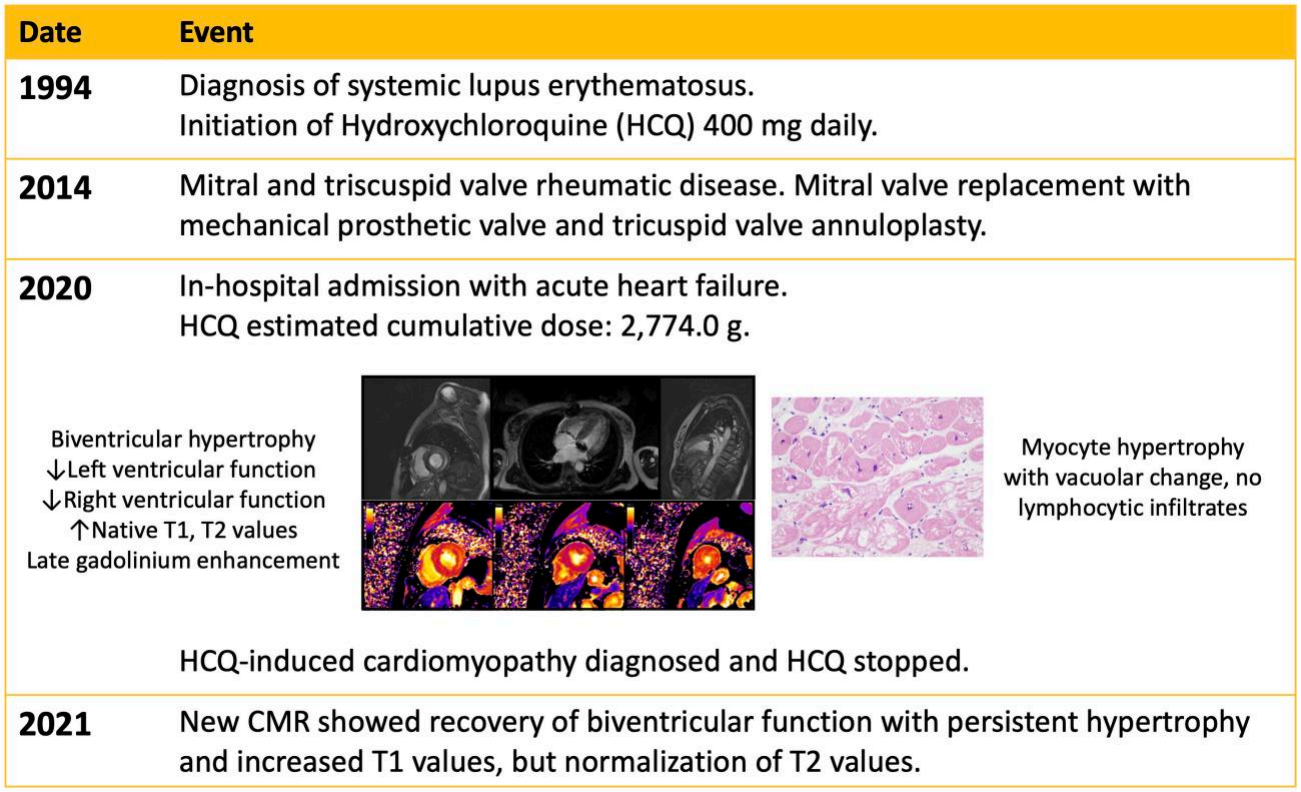


## Case presentation

A 45-year-old woman with a history of SLE presented to the Emergency Department for palpitations and shortness of breath within the last 2 weeks. Her medical history included rheumatic valve disease requiring mitral valve replacement with a mechanical prosthesis and surgical tricuspid valve annuloplasty 6 years earlier and a transient ischaemic attack 2 weeks earlier. Her medications included HCQ 400 mg/day (estimated total cumulative dose 2774 g), warfarin 49.5 mg/week, prednisone 7.5 mg/day, amiodarone 200 mg/day, colchicine 1 mg/day, and azathioprine 50 mg/day. Physical examination revealed normal prosthetic valve sounds and bilateral lung crackles. Chest X-ray showed pulmonary congestion. Laboratory results indicated elevated N-terminal pro-brain natriuretic peptide (peak value 16040 pg/mL) and high-sensitivity troponin I [peak value 597 ng/L, upper limit of normality (ULN) < 54], INR 2.7, and C-reactive protein 4.9 mg/L (ULN < 5.0 mg/L), with normal biomarkers of SLE activity. The electrocardiogram showed atrial fibrillation with rapid ventricular response and right bundle branch block (RBBB). The patient was admitted to the Cardiology Department with a diagnosis of acute heart failure (HF) and treated with high-dose furosemide.

A transthoracic echocardiogram (TTE) revealed severe concentric left ventricular (LV) hypertrophy, mildly reduced ejection fraction (EF) [left ventricular ejection fraction (LVEF) 51%] with global hypokinesia, and severe hypertrophy and systolic dysfunction of the right ventricle (RV) (see Supplementary material online, *Video S1*). A TTE performed 2 years before showed mild LV hypertrophy and normal LVEF.

A cardiac magnetic resonance (CMR) was performed with a Siemens Skyra 3T MRI scanner. CINE sequences (see Supplementary material online, *Video S2*) showed a non-dilated LV with concentric hypertrophy (mass 81 g/m^2^, septum 16 mm) and mildly depressed systolic function (LVEF 46%) with global hypokinesia, as well as a non-dilated RV with marked hypertrophy and depressed systolic function [right ventricular ejection fraction (RVEF) 38%]. No valvular insufficiencies or pericardial effusion were observed.

**Figure 1:**
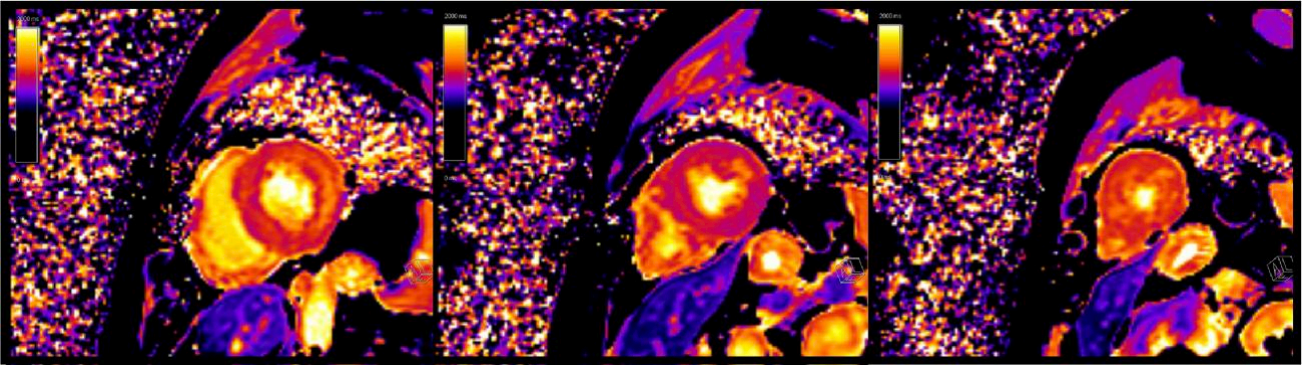
Elevated mean global myocardial native T1 value, with higher values in the basal segments of the inferior, inferolateral, and lateral walls and mid-lateral segment.

**Figure 2:**
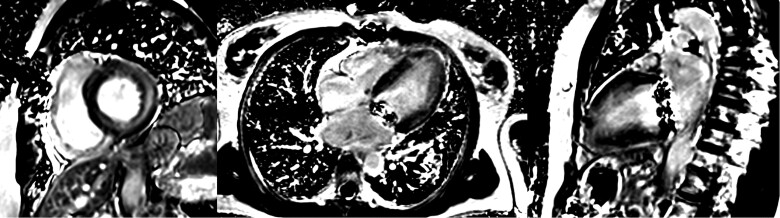
Mid-wall late gadolinium enhancement in the basal segments of the inferior, inferolateral, and lateral walls and mid-lateral segment of the left ventricle. Nodular enhancement foci in the apical segments of the left ventricle. Late gadolinium enhancement in the right ventricle.

Native T1 [MOLLI 5(3)3 sequence] was significantly increased in all myocardial segments (*Figure 1*); the maximal value was 1332 ms in the mid-septum (+7 SD, local ULN 1212 ms). T2 mapping (T2-flash sequence) showed uniformly elevated values (42–43 ms, local ULN 40 ms) with higher values on the basal lateral segment (46 ms). Late gadolinium enhancement (LGE) imaging showed extensive mid-wall enhancement in the basal inferior, basal inferolateral, basal lateral, and mid-lateral segments, as well as nodular enhancement foci in the apical segments of the LV and diffuse enhancement in the RV (*Figure 2*). Left ventricular extracellular volume was elevated (33%).

**Figure 3:**
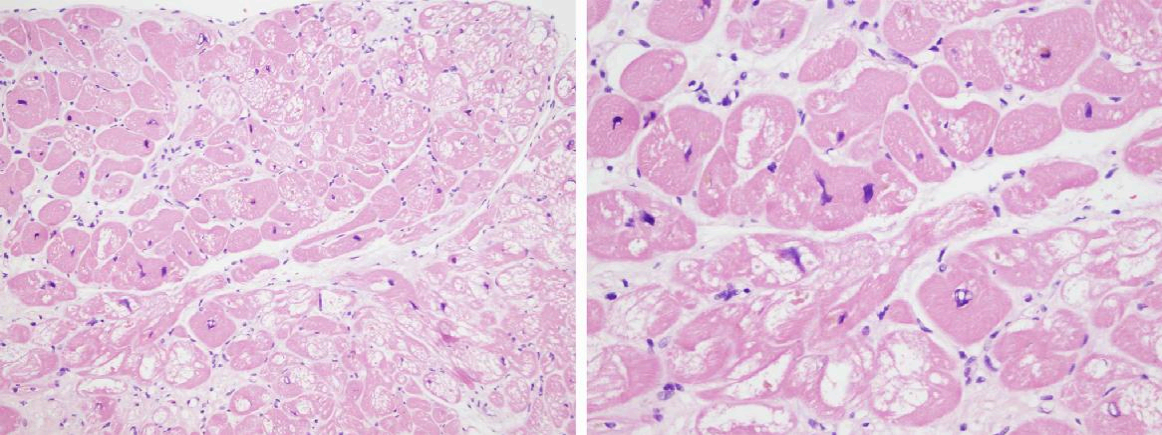
Optical microscope image showing myocyte hypertrophy with vacuolar change and absence of inflammatory infiltrates in light microscopy.

As CMR findings were suggestive of myocardial inflammation but did not allow differentiation between lupus myocarditis and HCQ myocardial injury, an endomyocardial biopsy (EMB) was performed. Light microscopy showed myocyte hypertrophy with vacuolar change and no lymphocytic inflammatory infiltrates (*Figure 3*). The diagnosis of myocardial HCQ toxicity was therefore confirmed. An electromyogram showed mild diffuse myopathy without signs of acute inflammatory injury. The ophthalmological examination ruled out HCQ-related retinopathy.

A multidisciplinary team which included internists, cardiologists, and rheumatologists decided to discontinue HCQ and leave the remaining immunosuppressive treatment unchanged, given the absence of signs of SLE activity. The response to the decongestion strategy was good, and the dose of diuretics was progressively reduced. The patient was discharged with a rate control strategy, therefore discontinuing amiodarone. Treatment upon discharge included previous medical treatment (except HCQ and amiodarone), bisoprolol 5 mg/day, digoxin 0.25 mg/day, and torasemide 2.5 mg/day.

During follow-up, clinical evolution was good, and the patient’s exercise capacity increased. A new CMR performed 1 year later showed LVEF (58%) and RVEF (49%) recovery (see Supplementary material online, *Video S3*) and normalization of T2 values (39 ms, ULN < 40 ms). However, biventricular hypertrophy and increased T1 (1370 ms, + 9SD) persisted. Late gadolinium enhancement imaging showed no changes compared with the previous CMR.

## Discussion

Hydroxychloroquine is a disease-modifying drug used in SLE. Long-term exposure results in drug accumulation and predisposes to adverse effects.^3^ Cardiac effects of HCQ include conduction abnormalities, myocardial thickening, restrictive cardiomyopathy, and HF. Cardiogenic shock and cardiac arrest have been reported.^4^ Risk factors for AMIC include older age, female sex, pre-existing cardiac disease, and kidney disease. The duration of use and cumulative dose appear to be decisive for disease development.^3,5^ Extracardiac toxicity can coexist (skin changes, retinopathy, and myopathy), but cardiac events can occur in the absence of other signs of toxicity.^6^ Differential diagnoses of AMIC include storage disorders (Fabry disease and Pompe disease), drug-induced myopathy, mitochondrial disorders, cardiac amyloidosis, or ischaemic heart disease.^3^ Diagnosis of AMIC remains challenging. Cardiac magnetic resonance provides useful information, but EMB is often needed. Cardiac magnetic resonance can guide biopsy sampling. Typical CMR findings include LV or biventricular hypertrophy, systolic dysfunction, hypokinesia, and mid-wall LGE.^6^ In a series of patients with SLE, patients with AMIC had higher T1 and T2 compared with healthy controls and lower T1 and T2 compared with patients with myocarditis. They also found that T1 increases with longer HCQ treatment among patients with AMIC.^7^

The typical findings on light and electron microscopy are explained by the pathophysiology of the disease. Hydroxychloroquine enters cardiac myocytes, binds to phospholipids, and inhibits phospholipases, causing an acquired lysosomal storage disorder. This leads to glycogen and phospholipid accumulation, resulting in concentric hypertrophy.^3^ Under light microscopy, the most consistent finding is the vacuolization of myocytes. Electron microscopy shows myeloid bodies, curvilinear bodies, and large secondary lysosomes.^8^

Our patient had been treated with HCQ for 19 years and showed signs of AMIC: RBBB, LV hypokinesia, and typical findings on CMR, i.e. biventricular hypertrophy, mid-wall LGE, and elevated T1, T2, and extracellular volume. Although glycosphingolipid accumulation seems to be the initial pathophysiological insult in AMIC, which could be detected with low native T1 values, in our case, native T1 values were strikingly increased. The prolonged exposure to HCQ may have led to diffuse fibrosis and myocardial oedema,^7^ indicated by increased T1 and T2 values. Indeed, the normalization of T2 12 months after withdrawal of HCQ could reflect stabilization of disease after HCQ discontinuation, similar to what is observed in AL amyloidosis when T2 values normalize after cessation of light chain deposition.^9^ However, though we observed biventricular systolic function recovery on follow-up CMR, hypertrophy and fibrosis persisted.

In conclusion, the diagnosis of AMIC is challenging. Although no specific sign of HCQ cardiotoxicity is available, CMR can rule out other conditions such as Fabry disease or ischaemic heart disease. Endomyocardial biopsy remains necessary in most cases to establish a definite diagnosis. Timely diagnosis is crucial to discontinue treatment with HCQ and prevent disease progression.

## Lead author biography


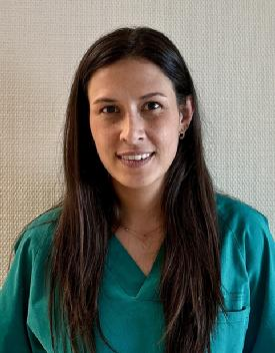


Lucía Cobarro, MD, is a cardiologist from Madrid, Spain. She graduated from the Autonomous University of Madrid in 2018. After completing her cardiology residency at La Paz University Hospital in May 2024, she's now specializing in cardiac electrophysiology as a fellow at 12 de Octubre University Hospital.

## Supplementary Material

ytae404_Supplementary_Data

## Data Availability

The data underlying this article are available in the article and its online Supplementary material. Further data will be shared on reasonable request to the corresponding author.
